# Cost and consequence analysis of the Healthy Choices at Work programme to prevent non-communicable diseases in a commercial power plant, South Africa

**DOI:** 10.4102/phcfm.v12i1.2217

**Published:** 2020-06-22

**Authors:** Darcelle D. Schouw, Robert Mash

**Affiliations:** 1 Department of Family and Emergency Medicine, Faculty of Medicine and Health Sciences, University of Stellenbosch, Cape Town, South Africa

**Keywords:** cost and consequence, incremental costs, risk factors, prevention, NCDs, workplace, LMIC

## Abstract

**Background:**

The workplace is an ideal setting for the implementation of a health promotion programmes to prevent non-communicable diseases (NCD). There are limited resources assigned to workplace health promotion programmes in low-and middle-income countries (LMIC).

**Aim:**

This study aimed to conduct a cost and consequence analysis of the Healthy Choices at Work programme.

**Setting:**

This study was conducted at a commercial power plant in South Africa.

**Methods:**

Incremental costs were obtained for the activities of the Healthy Choices at Work programme over a two-year period. A total of 156 employees were evaluated in the intervention, although the effect was experienced by all employees. An annual health risk factor assessment at baseline and follow up evaluated the consequences of the programme.

**Results:**

The total incremental costs over the two-year period accumulated to $4015 for 1743 employees. The cost per employee on an annual basis was $1.15 and was associated with a −10.2mmHg decrease in systolic blood pressure, −3.87mmHg in diastolic blood pressure, −0.45mmol/l in total cholesterol and significant improvement in harmful alcohol use, fruit and vegetable intake and physical inactivity (*p* < 0.001). There was no correlation between sickness absenteeism and risk factors for NCDs.

**Conclusion:**

The cost to implement the multicomponent HCW programme was low with significant beneficial consequences in transforming the workplace environment and reducing risks factors for NCDs. Findings of this study will be useful for small, medium and large organisations, the national department of health, and similar settings in LMICs.

## Introduction

Non-communicable diseases (NCDs), such as cardiovascular disease, cancer, chronic respiratory disease and diabetes, were responsible for 70% of global deaths in 2015, with more than three-quarters of these deaths occurring in low- and middle-income countries (LMICs).^1,2^ Risk factors for NCDs are greatly interconnected with environmental factors and increased urbanisation.^3^ In a study on 23 selected LMICs, chronic NCDs accounted for 50% of the gross burden of disease.^4^ The global increase of NCDs is of both epidemiological and economic concerns as they have a substantial impact on health, health services and organisational productivity.^4,5^ The World Economic Forum has identified chronic diseases as one of the most significant threats to global economic growth and estimated a cumulative loss to the global economy of $7 trillion between 2011 and 2025.^5,6^

In South Africa (SA), the burden of NCDs contributes to 57% of all deaths and is accompanied by significant impairments such as amputations, blindness, hemiparesis and speech problems.^7^ There is a substantial impact on the quality of life of individuals and families.^8,9^ The impact of NCDs is predicted to increase further in SA over the next decade.^10^ In SA, between 2006 and 2015, diabetes, stroke and coronary heart disease caused an estimated loss of $1.88 billion to the gross domestic product.^11^ Organisations are impacted by direct and indirect costs of high absenteeism and staff turnover as these diseases lead to morbidity in the working-age population, with obese workers costing organisations in the USA 49% more than their non-obese colleagues in terms of leave with pay.^12^ The direct costs include sick leave days, medical referrals and costs related to replacing absent employees, although indirect costs include losses from reduced productivity.^13^ Many of the deaths caused by NCDs are premature and occur in people of working age (36–64 years).^10^

Underlying these diseases are a number of behavioural risk factors such as tobacco smoking, harmful alcohol use, physical inactivity and an unhealthy diet.^14^ Interventions, therefore to prevent NCDs, focus on reducing harmful alcohol levels and tobacco smoking as well as improving physical activity and a healthy diet.^5^ Lifestyle behaviours are not just a matter of individual choice and control, but are also influenced by the environment in which individual decision-making is embedded.^15^

The World Health Organization (WHO) has identified the ‘best buys’ for LMICs to prevent and control NCDs.^16^ A ‘best buy’ is an intervention that is very cost-effective, feasible and culturally acceptable and adds on average an additional year of healthy life.^17^ According to WHO, the following should be considered when selecting such interventions: effectiveness, cost-effectiveness, affordability, capacity to implement, feasibility according to national circumstances, impact on health equity and its place within a menu of population-wide and individual interventions.^16^ Despite numerous potential interventions for the prevention and control of NCDs, choices regarding which interventions should be prioritised are critical, as resources in most countries are limited.

The SA National Department of Health, in its ‘Strategic Plan for the Prevention and Control of Non-Communicable Diseases 2013–2017’, promulgates a balance between individual-level and population-based strategies to prevent NCDs. Prevention and postponement of NCDs are more effective and less costly than treating those who become ill.^18^ NCDs in SA pose a challenge to economic development and there is a need for a priority-setting agency that assesses cost-effectiveness, accessibility and feasibility of diverse interventions.^19^ The national health budget does not include funding for robust health and costing data for NCDs to enable planning, budgeting and evaluation of activities.^19^

The workplace is an important setting for the prevention and control of NCDs.^20^ Workplace health promotion programmes (WHPPs) adopt a twofold approach that attributes a healthy lifestyle both to the individual’s responsibility and behaviour and to the influence of the environment that is outside the individual’s control.^21^ Organisations that implement WHPPs may subscribe to one or both approaches. The WHO’s ‘best buys’ found no conclusive cost-effectiveness analysis for interventions in the workplace, but nevertheless made some recommendations on the basis of the available evidence. These recommendations include (1) implementing nutrition education and counselling to increase fruit and vegetable uptake and (2) implementing multicomponent workplace physical activity programmes.^22^ In a systematic review, aligned to the WHO approach, 89% of LMICs had no studies on ‘best buys’. More than half of the studies reported effectiveness for group interventions in reducing tobacco, but found weaker evidence for interventions aimed at individuals. Because most of the LMICs have not conducted such research, consideration should be given to evaluate cost-effectiveness of interventions, while focusing on national priorities and interventions with the strongest evidence base.^23^ Recommended interventions for NCD risk factors in LMICs include making workplaces tobacco smoke-free with health information and warnings, reducing salt intake in food and implementing media campaigns on physical activity.^24^

There are relatively few studies showing the cost-effectiveness of WHPPs in Africa. Studies in Africa have shown that the NCD burden has increased and occurs concurrently with HIV, with workplace wellness programmes showing promise.^19^ However, the urban poor and unemployed have little access to these programmes. The families of the deceased carry the biggest brunt with two-thirds of poor households having no insurance for funeral costs and succumb to a loss of income from the deceased wage earners.^25^ In the USA, a WHPP with fire fighters showed a 10% reduction in cardiovascular events, but with an incremental cost-effectiveness ratio of $1.44 million per event prevented.^13^ A study evaluating the cost-effectiveness of a WHPP focusing on healthy eating in Ireland concluded that an organisational system-level dietary intervention was more cost-effective than individual education in terms of improved quality of life and reduced absenteeism.^26^ In a review of the economic impact of worksite interventions to promote healthy diet and physical activity, there was evidence of a 25% – 30% reduction in absenteeism and medical costs over 3–4 years.^27^ However, in a study of 44 UK worksites, exploring a WHPP designed to reduce physical inactivity, the intervention was not cost-effective.^28^

In many countries, including SA, policy focuses on occupational health and safety rather than on disease prevention in the workplace, and there is a lack of empirical evidence to support the cost-effectiveness of WHPPs.^29^ The literature on WHPPs in Africa has focused on short-term feasibility or pilot studies.^30^ Therefore, further research on the cost-effectiveness of interventions for the prevention of NCDs in the SA workplace is needed. The aim of this study therefore was to determine the incremental costs and consequences of the Healthy Choices at Work (HCW) in terms of improvement in risk factors for NCDs and sick leave absenteeism.

## Methods

### Study design

This study was an incremental cost and consequence study of the HCW programme at a commercial power plant in the Western Cape, SA.^31,32^ An incremental cost analysis was performed for additional costs incurred to the organisation resulting from the actions of the HCW. This study compared the incremental costs of implementing the HCW programme over 2 years with the consequences in terms of changes in risk factors for NCDs and sick leave.

### Setting

This study was conducted at a commercial power plant within a nature reserve close to Cape Town, SA. The 1743 permanent employees had a wide range of occupations, which included engineers, plant operators, physicists, technicians, artisans and support staff working in shifts. The power plant included a health and wellness department that conducted routine annual health risk assessments (HRAs) on self-selected employees and provided occupational health services. All staff members in the organisation were entitled to 180 days of sick leave over a 3-year cycle and were insured for medical care. The on-site health and wellness department conducted periodic medicals (physical examination, medical investigation of lung function, hearing, vision, heat stress and full blood counts on employees). Food was subsidised and provided by an external company which was contracted to operate the canteen and vending machines.

The power plant operated in a highly pressurised environment because of the lack of generation capacity in SA relative to the increased demand for electricity. Employees worked very intensively during outages when generation was halted for routine maintenance.

### The Healthy Choices at Work programme

The HCW intervention, which took place over 2 years, was implemented by participatory action research with a cooperative inquiry group (CIG) and is more fully described in a separate publication.^33^ A diverse CIG made up of 11 key decision-makers across the organisation (one financial manager, one wellness manager, one senior occupational health nurse, three engineering managers, two project management advisors, one industrial relations manager, one quality control officer and a manager from the organisation’s training department) led the intervention. The intervention focused on four key areas:

1. **Catering and the provision of food**: A new wellness meal (a low-fat, low-salt option with additional vegetables and fruit) was made available to employees on all shifts at no additional cost to them and was actively promoted to all workers via in-house daily newsflash articles, work team sessions and multimedia presentations. Healthy affordable snacks were also sold at the cafeterias, and fruit was provided as a healthy snack to employees working extended shifts.
2. **Opportunities for physical activity**: Areas were identified within the surrounding nature reserve for walking, running and cycling, and functional exercise classes were held four times a week. First Friday sports took place once a month and staff members were released from their duties to participate in 2.5 km/5 km walks, a 10 km run or a 25 km cycling. Employees were also encouraged to participate with their families in weekend park runs within their own communities.
3. **Provision of health and wellness services**: The HCW included two annual HRAs during the intervention. The HRAs provided feedback on NCD risk and the health and wellness team offered counselling to employees on behavioural change. Staff at the Health and Wellness department participated in a 3-day training course on brief behavioural change counselling using the 5 As (Ask, Alert, Assess, Assist and Arrange) approach in a guiding style to assist employees in making healthy lifestyle decisions.^34^
4. **Managerial buy-in and participation**: The management team consisted of an executive committee with 10–12 senior managers led by a general manager. Managers approved the prevention programme and led by example in choosing wellness meals, marketing activities, participating in physical activities and promoting health by broadcasting discussions of their own behavioural change.

### Evaluation of incremental costs

Incremental costs to the organisation that were associated with implementing the HCW were calculated for the 2-year period. Incremental costs were defined as additional costs that were incurred on top of existing expenditure. For example, allowing employees to participate in first Friday sports did not alter the cost of salaries and therefore these costs were not included, although the purchasing of additional exercise equipment was included. Research-related costs were excluded.

Activities that led to incremental costs were identified by the CIG for the four key areas of the intervention (catering, physical activity, health and wellness and management support). The information on costs was sourced from the organisation’s financial system by the finance manager and entered into an Excel spreadsheet.

These costs are reported in the ‘Results’ section according to the four main areas of intervention, which were further analysed in terms of the cost per capita and cost per annum.

### Evaluation of changes in risk factors for non-communicable diseases

A before-and-after study evaluated the changes in risk factors for NCDs, and the methods are fully reported elsewhere.^35^ The study population was all permanent employees working at the power plant, and there were no specific exclusion criteria. A sample of 156 employees was randomly selected. Sample size calculations confirmed the power of this sample to detect meaningful changes in risk factors for NCDs over time.^36^

Participants were assessed at baseline and 24 months by means of questionnaires, physical measurements and clinical tests performed by trained health professionals from the health and wellness department. The questionnaires included data on demographic information, medical and family history, medication use, diet, physical activity, alcohol consumption and psychosocial stress factors. The Global Physical Activity Questionnaire (GPAQ) was used to quantify levels of physical activity.^37^ Data on alcohol use were collected by using the validated Alcohol Use Disorders Identification Test (AUDIT) questionnaire.^38^ Questions on tobacco smoking, fruit and vegetable intake were extracted from the South African Demographic Health Survey Questionnaire.^39^ Questions on psychosocial stress at work or at home were obtained from the organisation’s in-house HRA questionnaire.

Clinical tests included blood pressure, blood glucose and total cholesterol, with physical measurements of body mass index, waist-to-hip ratio and a 10-year cardiovascular risk assessment. Blood pressure was measured by using standardised procedures for systolic and diastolic blood pressures.^40^ Point-of-care testing for random blood glucose and total cholesterol utilised a finger prick capillary blood sample.^41^ Standing height, weight and waist and hip circumferences were measured and body mass index and waist-to-hip ratio were calculated.^41^ A 10-year cardiovascular risk was assessed for each participant.^42^

Data were captured in an Excel spreadsheet and checked for errors or omissions. Data were then analysed using the Statistical Package for the Social Sciences (SPSS) Version 24.1, IBM, New York.

Metabolic equivalence of task metabolic equivalence of task (MET) minutes were calculated from the GPAQ data. A minimum of 600 MET minutes per week was required to be considered physically active. The AUDIT questionnaire included 10 questions with a 4-point Likert scale that gave a possible total score of 40. Respondents with a score of 7 or less were categorised as sensible drinkers, those with a score of 8–19 were categorised as potentially harmful drinkers and those with a score of 20 or more were categorised as potentially dependent drinkers. Descriptive analysis was used to calculate the mean and standard deviation or frequency and percentage of all variables at baseline and follow-up.

Paired *t*-tests were used to compare the mean differences for normally distributed numerical data (e.g. blood pressure, blood glucose, total cholesterol, body mass index, waist circumference, waist-to-hip ratio [WHR]) from baseline to follow-up at 2 years. McNemar’s chi-square test was used to compare paired binary categorical data from before to after (e.g. psychosocial stress, smoking, fruit and vegetables). Statistical significance was set at *p* < 0.05.

### Evaluation of changes in sick leave

Changes in sick leave related to NCDs were evaluated on the same sample of 156 employees as described above in the before-and-after study. Employee data on sick leave were collected on an annual basis by the Human Resources Information System. For this study, data were extracted on the study sample at baseline for the year prior to this study and at 2-year follow-up for the second year of the study intervention. Sick leave was measured in the following ways:

- The Gross Sickness Absentee Rate (GSAR) measured person days lost because of sick leave as a percentage of the total potential working days. The GSAR is always expressed as a percentage and is the international standard used for comparison of sickness absenteeism between companies, other work forces and countries.^43^ The ideal GSAR to aim for in SA is between 2% and 5%.^44^
- The absentee frequency rate (AFR) is the number of absence incidents per person for a given period and is calculated as the total number of absence incidents over a 12-month period divided by the number of employees over a 12-month period. Only the number of incidents and not the duration is calculated and a favourable AFR is less than 0.5.^44^

All data were captured on an Excel spreadsheet and checked for errors or omissions before analysis using IBM SPSS software version 25.1. Descriptive analysis was used to calculate the mean and standard deviation of GSAR and AFR before and after the intervention. Pearson’s correlation coefficient (*r*) test was conducted to investigate the relationship between GSAR and AFR for systolic blood pressure, diastolic blood pressure and total cholesterol, assuming that they were normally distributed. A one-way between-subjects analysis of variance (ANOVA) was conducted to compare the effect of fruit and vegetable intake and psychosocial stress on GSAR and AFR.

### Ethical consideration

Ethics approval was obtained from the Health and Research Ethics Committee (HREC) of Stellenbosch University (S15/08/165) and permission was obtained from the power plant to conduct this study. Written consent was obtained from each participant of the study.

## Results

### Incremental costs

Table 1 shows the incremental costs for the four key areas targeted in the intervention. The total incremental cost to the company was R59 183, which equated to an average of R29 591 per annum. The average incremental cost per employee for implementing the HCW was estimated as R33.95 ($2.3) over 2 years or R16.98 ($1.15) per annum.

**TABLE 1:** Incremental costs for activities.

Intervention	Cost for activities, 2016–2017 Rand	Cost for activities, 2016–2017 US $†
Opportunities for physical activity	31 583	2143
Catering and the provision of food	11 400	773
Provision of health and wellness services	16 200	1099
Managerial buy-in and participation	0	0

**Total**	**59 183**	**4015**

† , Exchange rate at 11 December 2019 of 1 Rand to US $0.068.

For physical activity, the incremental costs included the costs of equipment for functional exercise activities (e.g. mats, balls, skipping ropes) and the first Friday sports (e.g. colour powder). For catering activities, additional fruit was provided during outages. For health and wellness services the costs included the training of nine health professionals in brief behavioural change counselling. There were no incremental costs for the managers.

### Changes in risk factors

The mean age of the participants was 42.7 years (standard deviation [s.d.] = 9.7) and 64% were male. Table 2 presents the changes in behavioural and psychosocial risk factors for NCDs that were associated with the intervention. There were significant reductions in harmful alcohol use, physical inactivity and improved fruit and vegetable intake. There was no change in tobacco smoking. Participants reported significantly improved relationships with colleagues and self-perceived health that could possibly be attributed to the intervention. There were also significant improvements (Table 3) in mean systolic blood pressure (−10.2 mmHg), diastolic blood pressure (−3.87 mmHg) and total cholesterol (−0.45 mmol/L). There was no change in overweight, obesity or random glucose.

**TABLE 2:** Change in behavioural and psychosocial risk factors (*N* = 137).

Risk factors	Baseline %	Follow-up %	*p*
**Behavioural risk factors**			
Sensible alcohol drinker (AUDIT score < 8)	78.2	93.5	0.001
Harmful alcohol drinker (AUDIT score 8–19)	21.0	4.8	-
Dependent alcohol drinker (AUDIT score > 20)	0.8	1.6	-
Tobacco smoking	25.0	21.8	0.344
Inadequate fruit and vegetable intake (< 5 portions/day)	73.2	35.8	˂ 0.001
Insufficiently active (<600 MET minutes/week)	55.9	34.7	˂ 0.001
**Psychosocial factors**			
Relationship with colleagues	21.1	11.3	0.015
Lack of recognition	18.8	18.8	1.000
Lack of resources to do my work	29.5	30.3	1.000
Lack of meaningful work	15.1	13.5	0.664
Relationship with my supervisor	9.8	12.0	0.664
Lack of clarity concerning work outputs	20.9	15.7	0.265
Personal finances	29.8	18.3	0.008
My health or family member’s health	22.3	10.8	0.006
Relationship with family and children	16.0	13.7	0.690
Relationship with my partner or spouse	13.3	9.2	0.210
Emotional and mental health concerns	8.8	4.7	0.227
I have challenges with addictions	3.3	0.8	0.250

AUDIT, Alcohol Use Disorders Identification Test; MET, metabolic equivalence of task.

**TABLE 3:** Change in metabolic risk factors (*N* = 137).

Risk factors	Baseline		Follow-up		Mean of the difference	95% CI	*p*
	Mean	s.d.	Mean	s.d.			
Systolic blood pressure (mmHg)	131.6	18.5	121.4	14.6	−10.2	−7.3: −13.2	< 0.001
Diastolic blood pressure (mmHg)	83.4	13.7	79.5	8.8	−3.87	−1.8: −5.8	˂ 0.001
Total cholesterol (mmol/L)	5.6	1.1	5.1	1.1	−0.45	−0.3: −0.6	˂ 0.001
Random glucose (mmol/L)	5.7	1.5	6.0	2.0	0.31	−0.6: 0.2	0.069
Body mass index (kg/m^2^)	29.0	5.5	29.0	5.7	−0.05	−0.4: 0.3	0.760
Waist circumference (cm)	92.1	14.3	92.2	14.4	0.05	−1.1: 1.0	0.926
Waist-to-hip ratio (cm)	0.86	0.1	0.87	0.1	−0.00	−0.0: 0.0	0.484

s.d., standard deviation; CI, confidence interval.

### Changes in sick leave

Tables 4 and 5 show the relationship between changes in sick leave and changes in risk factors. Overall, there was no meaningful correlation between sick leave and risk factors, although a decrease in alcohol intake was weakly associated with an increase in sick leave.

**TABLE 4:** Relationship of sick leave and categorical risk factors.

Risk factors	Mean change in GSAR	s.d.	*p*	Mean change in AFR	s.d.	*p*
**Change in fruit and vegetable intake**						
Increase in intake (*n* = 29)	−0.67	2.9	0.017	−0.52	1.9	0.892
No change in intake (*n* = 55)	0.51	4.4		−0.25	3.0	
Decrease in intake (*n* = 9)	−1.0	3.1		−0.56	2.8	
**Change in perceived personal and family health**						
Increase in stress (*n* = 4)	0.95	0.8	0.918	−1.75	2.9	0.087
No change in stress (*n* = 73)	−0.16	4.8		−0.23	2.8	
Decrease in stress (*n* = 16)	−0.86	3.4		−0.37	2.3	
**Change in relationship with colleagues**						
Increase in stress (*n* = 4)	−1.99	3.1	0.711	0.50	1.7	0.552
No change in stress (*n* = 68)	−0.10	3.7		−0.03	2.6	
Decrease in stress (*n* = 19)	−2.13	7.1		−1.53	3.0	

GSAR, gross sickness absentee rate; s.d., standard deviation; AFR, absentee frequency rate.

**TABLE 5:** Correlation of sick leave with numerical risk factors.

Correlation	Pearson correlation (*r*)	*p*
GSAR vs. systolic blood pressure	0.171	0.096
GSAR vs. diastolic blood pressure	−0.102	0.325
GSAR vs. total cholesterol	−0.168	0.101
GSAR vs. AUDIT	−0.258	0.011
GSAR vs. METS	−0.048	0.642
AFR vs. systolic blood pressure	−0.087	0.399
AFR vs. diastolic blood pressure	−0.160	0.118
AFR vs. total cholesterol	−0.154	0.135
AFR vs. AUDIT	−0.259	0.011
AFR vs. METS	−0.002	0.981

GSAR, gross sickness absentee rate; AUDIT, Alcohol Use Disorders Identification Test; METS, metabolic equivalence of task; AFR, absentee frequency rate.

## Discussion

The HCW was associated with significant improvements in risk factors for NCDs that are likely to be clinically meaningful. This study also suggests that the HCW is a highly affordable intervention. Significant improvement in risk factors for NCDs was seen with minimal incremental costs to the company. The cost to implement the intervention was approximately $1 per individual per annum, which is important for LMICs where resources are constrained. The WHO ‘best buys’ approach defined a number of criteria to assess interventions, and these are used to structure the discussion: affordability, capacity to implement, feasibility according to national circumstances, impact on health equity and the need to implement a combination of population-wide policy interventions and individual interventions.^5^ The HCW programme has the potential to be considered a ‘best buy’ when assessed according to these criteria.

### Affordability

The implementation of HCW was very affordable when compared with other types of interventions. For example, using the Internet as a vehicle for health promotion to impact physical activity as well as fruit and vegetable intake was more costly ($425 per person) and not effective.^45^ The cost to implement a system-level dietary modification intervention to reduce absenteeism in the workplace was $56 per employee per annum.^26^

Programmes such as HCW that focus primarily on environmental modification rather than education from health professionals in the workplace are more likely to be affordable.^23^ The HCW programme also appears more affordable than some community-based interventions for reduction in systolic blood pressure ($62 per person for a 1 mmHg decrease versus $1 per person for a 10 mmHg decrease), especially as the HCW cost was not limited to an effect on blood pressure alone.^46^

### Capacity to implement

Sufficient and trained capacity was provided in the form of a dedicated team of professionals from the organisational health and wellness department as well as from the operational sectors in the organisation and CIG. The alcohol policy and subsidised policy on providing wellness meals was incorporated in the organisational regulations. Future capacity can be increased by incorporating a train-the-trainer programme whereby volunteers within the organisation are invited as health and wellness champions and trained on how to implement WHPP. An example of such a programme is the Work@Health T3 Programme, an evidence-based curriculum whereby employees and contract staff are trained in health promotion to train other employees. The Work@Health programme is effective in that the curriculum can be adapted to the context and culture of the organisation and therefore build on the internal capacity to sustain health promotion in the workplace.^47^

### Feasibility according to national circumstances

This study shows the feasibility of utilising currently available resources to relieve the burden of NCDs amongst employees. However, the HCW relied on significant indirect costs (extended time given off for participation in sport, staff within the organisation rendering the necessary services, clinical testing, media and advertising and occupational health services) made possible by the commitment of the senior management team of the organisation.

### Impact on health equity of interventions

Inequalities in health status were indirectly addressed as the whole organisation was open to participate in the activities and enjoyed the benefits of the HCW programme. In broader terms, the HCW programme contributed to improving health equity for permanent and contract staff by partnering with community and government organisations to participate in additional wellness activities and receive education on NCDs. Health equity was a leader-driven priority, whereby all staff members were encouraged to participate in activities, irrespective of their employment status within the organisation. However, full equity was not afforded to contract workers as they did not have the same advantages of permanent employees (access to private medical insurers, time off incentives and access to healthcare facilities on the commercial plant).

### The need to implement a combination of population-wide policy interventions and individual interventions

The HCW programme itself targeted the whole workplace-based population and not just individuals in its systematic changes to the environment, although it also included individual-level interventions such as behavioural change counselling. Implementation of WHPPs of this nature throughout SA workplaces could contribute to policy interventions that target the employed population.

Although we were unable to calculate an incremental cost-effectiveness ratio for the HCW, we believe that given the very low cost it is likely to meet the WHO best buy criteria of < $100/disability adjusted life year (DALY) for LMICs.^16^ If additional funding can be obtained, such a calculation will be possible.^47^

There was no relationship between the HCW programme and the reduced sick leave. Absenteeism because of illness may have been influenced by many different factors, which could mask any impact of the HCW on sickness from NCDs. The time frame may be too short to determine the impact of the HCW on NCDs and there are complications such as cardiovascular events. As the impact of the HCW will only be felt years later, the HCW needs further evaluation to determine effectiveness on absenteeism. The correlation between a reduction in harmful alcohol use and an increase in sick leave was unexpected. The reduction in alcohol use was attributed to the HCW intervention, and it is difficult to explain why this would lead to an increase in sick leave. Elsewhere a similar phenomenon has been noted, but attributed to a link between reduction in alcohol intake and the development of other illnesses.^48^ Other studies found a U-shaped relationship between alcohol consumption and sickness absenteeism such that people who abstained from alcohol had higher sickness absenteeism than people who consumed alcohol moderately.^49^

## Limitations

Although the incremental costs and consequences have been compared in this study, it would have been helpful to calculate an incremental cost-effectiveness ratio. A mini Markov model has been developed for the South African context to assess the incremental cost effective ratio (ICER) for interventions on risk factors for NCDs. Unfortunately the model is available only in the United States, and additional funds would be needed to analyse the data. This study did not measure indirect costs (salaries, treatment, clinical tests, travelling, reimbursements, catering, devices, etc.) already paid for by the organisation or costs associated with NCDs borne by the employee.

The before-and-after study design cannot prove the effectiveness of the HCW per se but has allowed the researcher to measure changes in risk factors associated with the intervention. The whole organisation was exposed to the intervention, which made the selection of a control group difficult. Improvements in risk factors could be because of other confounding factors, although prior to the intervention the annual HRAs suggested a progressive increase in risk as retrieved from employee medical records.

## Recommendations

- As the HCW appears to be cost-effective, the programme could be implemented in other medium and large enterprises, which have similar organisational settings to potentially deliver the programme.
- Further evidence of cost-effectiveness should be obtained from experimental study designs that include full cost-effectiveness analysis and measurement of the impact on productivity.
- The low-cost and beneficial consequences of the HCW support the inclusion of such WHPPs in the National Department of Health’s policy on NCD prevention and control.

## Conclusion

This study has demonstrated low incremental costs and substantial beneficial consequences in terms of risk factors for NCDs in the HCW programme. Despite reductions in risk factors, there was no reduction in sick leave. This study supports the value of WHPPs in the SA policy context for similar large and moderate enterprises to reduce the risk of NCDs. Future studies should formally measure the incremental cost-effectiveness ratio and also assess the effect on productivity.
